# Bayesian semiparametric regression models to characterize molecular evolution

**DOI:** 10.1186/1471-2105-13-278

**Published:** 2012-10-30

**Authors:** Saheli Datta, Abel Rodriguez, Raquel Prado

**Affiliations:** 1, Fred Hutchinson Cancer Research Center, Seattle, WA, USA; 2 Department of Applied Mathematics and Statistics, University of California Santa Cruz, Santa Cruz, CA, USA

## Abstract

**Background:**

Statistical models and methods that associate changes in the physicochemical properties of amino acids with natural selection at the molecular level typically do not take into account the correlations between such properties. We propose a Bayesian hierarchical regression model with a generalization of the Dirichlet process prior on the distribution of the regression coefficients that describes the relationship between the changes in amino acid distances and natural selection in protein-coding DNA sequence alignments.

**Results:**

The Bayesian semiparametric approach is illustrated with simulated data and the abalone lysin sperm data. Our method identifies groups of properties which, for this particular dataset, have a similar effect on evolution. The model also provides nonparametric site-specific estimates for the strength of conservation of these properties.

**Conclusions:**

The model described here is distinguished by its ability to handle a large number of amino acid properties simultaneously, while taking into account that such data can be correlated. The multi-level clustering ability of the model allows for appealing interpretations of the results in terms of properties that are roughly equivalent from the standpoint of molecular evolution.

## Background

The structural and functional role of a codon in a gene determines its ability to freely change. For example, nonsynonymous (amino acid altering) substitutions may not be tolerated at certain codon sites due to strong negative selection, while at other sites some nonsynonymous substitutions may be allowed if they do not affect key physicochemical properties associated with protein function [1]. Thus, at such preferentially changing sites, more frequent substitutions occur between physicochemically similar amino acids (or codons which lead to those amino acids) than dissimilar ones [2-4]. Methods which use changes in physicochemical amino acid properties have thus been proposed in the study of evolution. For example, [5-7] use distances to calculate deviations from neutrality for a particular amino acid property. Alternative approaches model the evolution of protein coding sequences as continuous-time Markov chains with rate matrices that distinguish between property-altering and property-conserving mutations as in [8] and [9]. More recently, [10] proposed a Bayesian hierarchical regression model that compares the observed amino acid distances to the expected distances under neutrality for a given set of amino acid properties and incorporates mixture priors for variable selection. The hierarchical mixture priors enable the model in [10] to identify neutral, conserved and radically changing sites, while automatically adjusting for multiple comparisons and borrowing information across properties and sites.

A common feature of all the methods listed above is the implicit assumption that properties are independent from each other in terms of their effect on evolution. A review of the amino acid index database (available for example at http://www.genome.jp/dbget/aaindex.html), which lists more than 500 amino acid properties, shows that a large number of them are highly correlated. Although the correlations we observe in the data can be different from those computed from the raw amino acid scores due to the influence of factors such as codon bias, by ignoring these correlations we are also ignoring the fact that correlated properties may affect a particular site in similar ways. Hence, approaches that do not take into account the correlations in the rates of mutations on different codons do not make use of key information about the relative importance of different physicochemical properties on molecular evolution.

A natural way to account for correlations in the data is by considering a factor structure, see for example [11]. However, selecting the number and order of the factors can be a difficult task in this type of factor models. In addition, the particular structure of the model in [11] makes it difficult to incorporate the effect of the factors on regions that are very strongly conserved. This paper extends the Bayesian hierarchical regression model in [10] by placing a nonparametric prior on the distribution of the regression coefficients describing the effect of properties on molecular evolution. The prior is an extension of the well known Dirichlet process prior [12,13] to model separately exchangeable arrays [14,15]. As in [10], the main goal of the model described in this paper is to identify sites that are either strongly conserved or radically changing. In order to account for correlations across properties, our model clusters properties with similar effects on evolution, and within each such group, clusters sites with similar regression coefficients and nonparametrically estimates their distribution. In addition to accounting for correlations across properties, this structure allows us to dramatically reduce the number of parameters in the model and generate interpretable insights about molecular evolution at the codon level.

Although the clusters of properties can in principle be considered nuisance parameters that are of no direct interest, in practice posterior inference on the clustering structure can provide interesting insights about the molecular evolution process of a given gene. Indeed, as will become clear in the following sections, our approach incorporates the effect of amino acid usage bias. Hence, any significant differences between the cluster structure estimated from the observed protein-coding sequence alignment and the correlation structure derived from the raw distances between the properties in such cluster can be interpreted a signal of extreme amino acid usage bias in that particular region of the genome.

The rest of the paper is organized as follows. A brief review of DP mixture models along with the details of our model is provided in the Methods section. This section also includes a review of some of the currently available methods for characterizing molecular evolution that take into account changes amino acid properties. The model is then evaluated via simulation studies and illustrated through a real data example. The simulated and real data analyses, as well as comparisons between the proposed semiparametric regression approach and other methods, are presented in Results and discussion. Finally, the Conclusions section provides our concluding remarks.

## Methods

### Dirichlet process mixture models

The Dirichlet process (DP) was formally introduced by [12] as a prior probability model for random distributions *G*. A DP(*ρ*, *G*_0_) prior for *G* is characterized by two parameters, a positive scalar parameter *ρ*, and a parametric base distribution (or centering distribution) *G*_0_. *ρ* can be interpreted as the precision parameter, with larger values of *ρ*resulting in realizations of *G* that are closer to the base distribution *G*_0_.

One of the most commonly used definitions of the DP is its constructive definition [13], which characterizes DP realizations as countable mixtures of point masses. Specifically, a random distribution *G* generated from DP(*ρ*, *G*_0_) is almost surely of the form

$$G(\cdot )=\overset{\infty }{\underset{l=1}{\sum }}w_{l}\delta_{\phi_{l}}(\cdot ),$$

where $\left(\delta_{\phi_{l}}(\cdot )\right)$ denotes a point mass at *ϕ*_*l*_. The locations *ϕ*_*l*_ are i.i.d. draws from *G*_0_, while the corresponding weights *w*_*l*_ are generated using the following “stick-breaking” mechanism. Let *w*_1_=*v*_1_and define $\left(w_{l}=v_{l}\overset{l-1}{\underset{r=1}{\prod }}(1-v_{r})\right)$ for *l*=2,3,…, where {*v*_*l*_:*l*=1,2,…} are i.i.d. draws from a Beta(1, *ρ*) distribution. Defining the weights in this way ensures $\left(\overset{\infty }{\underset{l=1}{\sum }}w_{l}=1\right)$. Furthermore, the sequences {*v*_*l*_:*l*=1,2,…} and {*ϕ*_*l*_:*l*=1,2,…} are independent.

The DP is most often used to model the distribution of random effects in hierarchical models. In the simplest case where no covariates are present, these models reduce to nonparametric mixture models (e.g., [16-18]). Assume that we have an independent sample of observations *y*_1_*y*_2_,…,*y*_*n*_ such that $\left(y_{i}|\theta_{i}\overset{\mathrm{ind}}{∼}k(\cdot ;\theta_{i})\right)$, where *k*(·;*θ*_*i*_) is a parametric density. Then, the DP mixture model places a DP prior on *θ*_*i*_ as

$$\begin{array}{lll} \theta_{i}|G\;\overset{\mathrm{i.i.d.}}{∼} & \;G,\;i=1,\ldots ,n\; & \;\\ G|\rho \;∼ & \;\text{DP}(\rho ,G_{0})\; & \; \end{array}$$

The almost sure discreteness of realizations of *G* from the DP prior allows ties in *θ*_*i*_, making DP mixture models appealing in applications where clustering is expected. The clustering nature is easier to see from the Pólya urn characterization of the DP [19] which gives the induced joint distribution for the *θ*_*i*_s, by marginalizing *G* over its DP prior. Under that representation, we can write $\left(\theta_{i}=\theta_{\xi_{i}}^{*}\right)$ where $\left(\theta_{1}^{*},\theta_{2}^{*},\ldots \right)$ is an independent and identically distributed sample from *G*_0_ and the indicators *ξ*_1_,…,*ξ*_*n*_ are discrete indicators sequentially generated with *ξ*_1_=1 and

$$\text{Pr}(\xi_{i+1}=k|\rho ,\xi_{i},\ldots ,\xi_{1})=\left\{\begin{array}{c} \frac{r_{k}^{i}}{i+\rho }\;k\leq \underset{j\leq i}{\max}\{\xi_{i}\}\\ \frac{\rho }{i+\rho }\;k=\underset{j\leq i}{\text{max}}\{\xi_{i}\}+1, \end{array}\right)$$

where $\left(r_{k}^{i}=\overset{i}{\underset{j=1}{\sum }}I(\xi_{j}=k)\right)$ and

$$I(\xi_{j}=k)=\left\{\begin{array}{l} 1\;\xi_{j}=k\\ 0\;\text{otherwise.} \end{array}\right)$$

One advantage of DP mixture models over other approaches to clustering and classification is that they allow us to automatically estimate the number of components in the mixture. Indeed, from the Pólya urn representation of the process it should be clear that, although the number of *potential* mixture components is infinite, the model implicitly places a prior on the number of components that, for moderate values of *ρ*, favors the data being generated by an effective number of components *K*^∗^=max_*i*≤*n*_{*ξ*_*i*_}<*n*.

### The model

Our data consist of observed and expected amino acid distances derived from a DNA sequence alignment, a specific phylogeny, a stochastic model of sequence evolution, and a predetermined set of physicochemical amino acid properties. In the analyses presented here, we disregard uncertainty in the alignment/phylogeny/ancestral sequence level since our main focus is the development and implementation of models that allow us to make inferences on the latent effects that several amino acid properties may have on molecular evolution for a given phylogeny and an underlying model of sequence evolution. Extensions of these analyses that take into account these uncertainties are briefly described in Conclusions. For further discussion on this issue, see also [10].

In order to calculate the observed distances, we first infer the ancestral sequences under a specific substitution model and a given phylogeny. In our applications, we use PAML version 3.15 [20] and the codon substitution model of [21], which accounts for the possibility of multiple substitutions at a given site. Nonsynonymous substitutions are then counted by comparing DNA sequences between two neighboring nodes in the phylogeny. The observed mean distance, denoted as *y*_*i*,*j*_ for site *i* and property *j*, is obtained as the mean absolute difference in the property scores due to all nonsynonymous substitutions at site *i*. Only those sites with at least one nonsynonymous change from the ancestral level are retained for further analysis.

To compute the expected distances, note that each codon can mutate to one of at most nine alternative codons through a single nucleotide substitution [5], only some of which are nonsynonymous (changes to stop codons are ignored). Let *N*_*k*_ be the number of nonsynonymous mutations possible through a single nucleotide change, corresponding to a particular codon *k* (*k*=1,…,61). Let $\left(D_{k,l}^{i,j}\right)$ be the absolute difference in property *j* between nonsynonymous codon pairs at site *i* differing at one codon position, where *l*=1,…,*N*_*k*_. The frequency of codon *k* at a particular site *i* in the DNA sequence under study is denoted by $\left(F_{k}^{i}\right)$. Then, the expected mean distance for a particular site *i* and a given property *j* is given by

$$x_{i,j}\equiv D_{E}^{i,j}=\frac{\overset{61}{\underset{k=1}{\sum }}F_{k}^{i}\overset{N_{k}}{\underset{l=1}{\sum }}D_{k,l}^{i,j}}{\overset{61}{\underset{k=1}{\sum }}F_{k}^{i}N_{k}}.$$

We consider a hierarchical regression model that relates *x*_*i*,*j*_ to *y*_*i*,*j*_and allows us to compare the expected and observed distances at the codon level for several properties simultaneously with the following rationale. If a given site *i* is neutral with respect to property *j*, then *y*_*i*,*j*_≈*x*_*i*,*j*_. If property *j* is conserved at site *i*, then *y*_*i*,*j*_<<*x*_*i*,*j*_ and finally, if property *j* is radically changing at site *i*, then *y*_*i*,*j*_>>*x*_*i*,*j*_.

To construct our model, we first standardize the distances *x*_*i*,*j*_ and *y*_*i*,*j*_ by dividing them by the maximum possible distance for each property. This enables us to use priors with the same scale for all the regression coefficients. Our regression model for the standardized distances $\left(y_{i,j}^{*}\right)$ and $\left(x_{i,j}^{*}\right)$, for sites *i*=1,…,*I* and properties *j*=1,…,*J*, can be written as

(1) $$y_{i,j}^{*}|\beta_{i,j},\sigma_{i,j}^{2}∼\left\{\begin{array}{c} \text{N}(\beta_{i,j}x_{i,j}^{*},\sigma_{i,j}^{2})\;\;\;\;\;\text{if}\;\;\beta_{i,j}=0\\ \text{N}(\beta_{i,j}x_{i,j}^{*},\sigma_{i,j}^{2}/n_{i}^{O})\;\text{if}\;\;\beta_{i,j}\neq 0, \end{array}\right)$$

where $\left(n_{i}^{O}\right)$ is the observed number of nonsynonymous changes at a particular site *i* and *β*_*i*,*j*_ and $\left(\sigma_{i,j}^{2}\right)$ are the regression coefficient and variance parameter associated with site *i* and property *j*. The mixture model accounts for the fact that some of the $\left(y_{\mathrm{ij}}^{*}\right)$s can be equal to zero as some nonsynonymous changes do not change the value of the property being measured (e.g., Aspargine, Aspartic acid, Glutamine, Glutamic acid all have the same hydropathy score).

To complete the model, we need to describe a model for the matrix of regression coefficients *β*_*i*,*j*_. There are a number of possible models for this type of data which utilize Bayesian nonparametric methods; some recent examples include the infinite relational model (IRM) [22,23], the matrix stick breaking process (MSBP) [24], and the nested infinite relational model (NIRM) [14,15].

**Figure 1:**
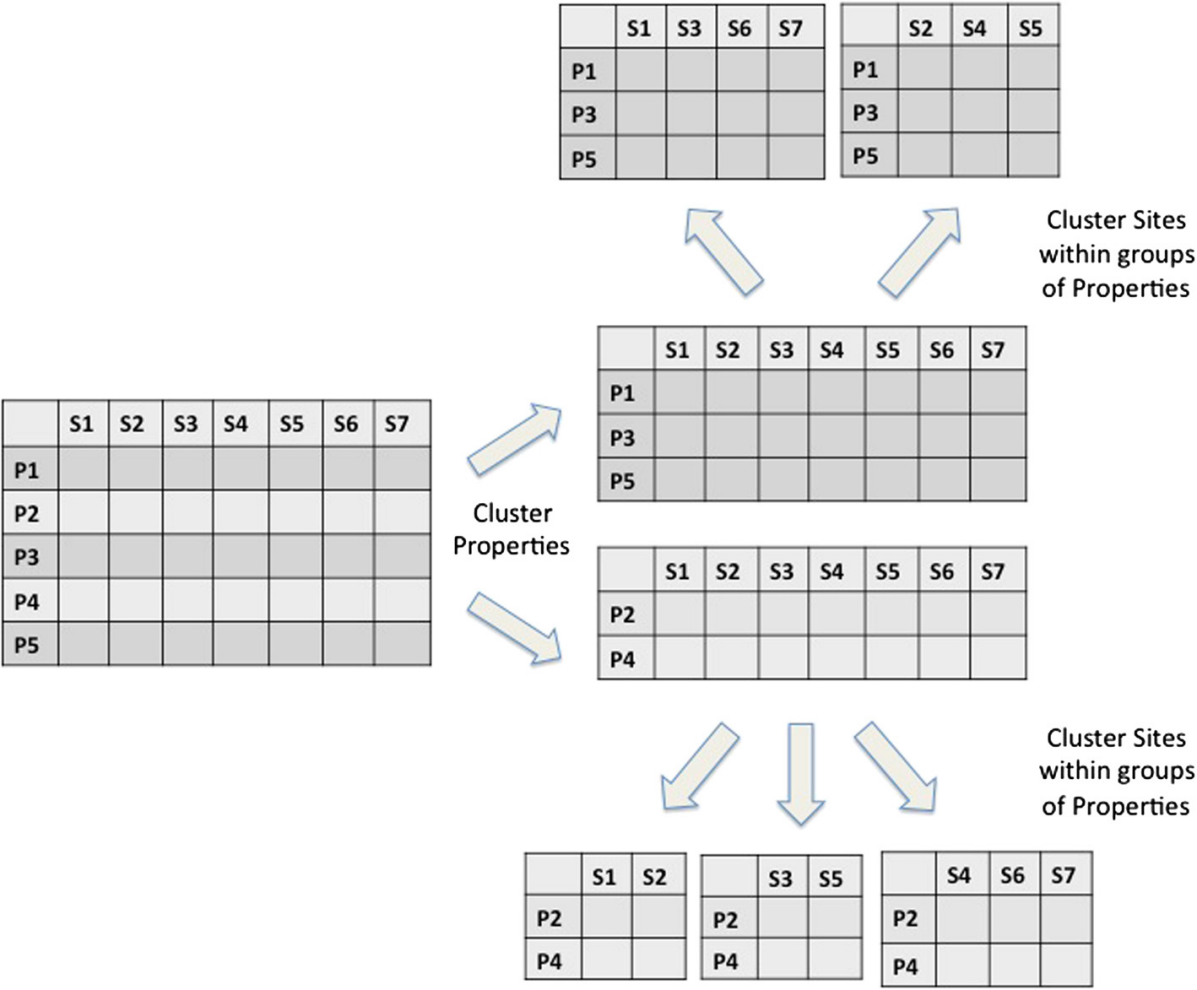
**Stylized representation of our model.** Each sub table at the second level of clustering shares a common value for the regression coefficient *β*_*i*,*j*_. Rows correspond to properties, while columns correspond to sites.

In this paper we focus on the NIRM, which is constructed by partitioning the original matrix into groups corresponding to entries with similar behavior. This is done by generating partitions in one of the dimensions of the matrix (say, rows) that are nested within clusters of the other dimension (columns). This structure allows us to identify groups of (typically correlated) properties with similar pattern and then, within each such group, identify clusters of sites with similar values of *β*_*i*,*j*_(Figure 1 provides a graphical representation of this idea). In our setting, we take $\left(\left[\theta_{\mathrm{ij}}]=[\beta_{i,j},\sigma_{i,j}^{2}\right]\right)$ and employ a NIRM to generate a prior for [***θ***_*ij*_].

More specifically, we denote by ***θ***_*j*_=(*θ*_1,*j*_,…,*θ*_*Ij*_)^*′*^the vector of regression coefficients and the associated variances corresponding to property (column) *j*. To obtain clusters for the properties, we assume that ***θ***_*j*_∼*F*, where

(2) $$F=\overset{\infty }{\underset{k=1}{\sum }}\Pi_{k}\delta_{\theta_{k}^{*}}$$

is a random distribution such that $\left(\Pi_{k}=v_{k}\underset{s<k}{\prod }(1-v_{s})\right)$, *v*_*k*_∼Beta(1,*ρ*), and $\left(\theta_{k}^{*}∼H_{k}\right)$. Indeed, the discrete nature of *F* ensures that ties among the ***θ***_*j*_happen with non-zero probability.

To obtain cluster-specific partitions for the sites (rows), *H*_*k*_ (the joint distribution associated with all sites for a given cluster of properties) has to be chosen carefully. In particular, we write $\left(\theta_{k}^{*}={\left(\theta_{1,k}^{*},\ldots ,\theta_{I,k}^{*}\right)}^{\prime }\right)$ for any specific specific cluster of properties *k* and let

(3) $$\theta_{i,k}^{*}∼\overset{\infty }{\underset{l=1}{\sum }}w_{l,k}\delta_{\varphi_{l,k}},$$

with $\left(w_{l,k}=u_{l,k}\underset{r<l}{\prod }\{1-u_{r,k}\}\right)$, *u*_*l*,*k*_∼Beta(1,*γ*_*k*_) for every *k*, and *φ*_*l*,*k*_ are independently drawn from the baseline measure *G*_0,*l*,*k*_.

The baseline measure *G*_0,*l*,*k*_ is chosen to accommodate the fact that some $\left(y_{i,j}^{*}\right)$s can be zero, since some nonsynonymous changes can keep the value of the property being measured unchanged. Thus, *G*_0,*l*,*k*_is a mixture with a point mass at zero and a continuous density otherwise. To allow for a more flexible model we assume that different prior variances are associated with the $\left(y_{i,j}^{*}\right)$s which are zero and those $\left(y_{i,j}^{*}\right)$s that are different from zero, with the specific form of *G*_0*lk*_as below.

(4) $$\varphi_{l,k}=(\phi_{l,k},\vartheta_{l,k}^{2})|G_{0\mathrm{lk}}∼G_{0\mathrm{lk}}$$

with

$$G_{0\mathrm{lk}}=\lambda 1_{\left\{\phi_{l,k}=0\right\}}p_{1}(\vartheta_{l,k}^{2})+(1-\lambda )p(\phi_{\mathrm{lk}}|\vartheta_{l,k}^{2})p_{2}(\vartheta_{l,k}^{2}),$$

where $\left(p_{1}(\vartheta_{l,k}^{2})∼\right)$Inv-Ga(*a*_*κ*_,*b*_*κ*_), $\left(p(\phi_{l,k}|\vartheta_{l,k}^{2})∼\text{N}(\alpha_{k},\vartheta_{l,k}^{2}/V_{0})\right)$ and $\left(p_{2}(\vartheta_{l,k}^{2})∼\right)$Inv-Ga$\left(\left(a_{\sigma }^{*},b_{\sigma }^{*}\right)\right)$. Here *ϕ*_*l*,*k*_ and $\left(\vartheta_{l,k}^{2}\right)$ respectively denote the unique values *β*_*i*,*j*_and $\left(\sigma_{i,j}^{2}\right)$ can take, whereas *λ* is the prior probability that *ϕ*_*l*,*k*_has the value zero (i.e., the properties associated with this cluster are strongly conserved at this cluster of sites).

Note that our model implies that both sites and properties are exchangeable a priori. If no additional prior information is available, this type of assumption seems reasonable. However, a posteriori, it is possible to have sites behave differently in different clusters.

To complete the model we place hyperpriors on all parameters of the resulting model. Conjugate priors are chosen for ease of computation. *α*_*k*_ denotes the mean for the *ϕ*_*l*,*k*_s that are different from zero belonging to a specific cluster of properties *k* and is assumed to have a N(*m*_*α*_,*C*_*α*_) prior for all *k*. The DP concentration parameters *ρ*and *γ*_*k*_ are assumed to follow Ga(*a*_*ρ*_,*b*_*ρ*_) with mean *a*_*ρ*_/*b*_*ρ*_, and Ga(*a*_*γ*_,*b*_*γ*_) with mean *a*_*γ*_/*b*_*γ*_ for all *k*, respectively. *λ*, which is the prior probability for the point mass at 0 in *G*_0*lk*_, follows a Beta(*a*_*λ*_,*b*_*λ*_). The specific choice of hyperparameters is discussed later as part of each data analysis. In general, we use Ga(1,1) priors for the DP concentration parameters and a N(1,*C*_*α*_) prior for *α*_*k*_to correspond to our assumption of neutrality a priori for the properties.

### Related work

We compare results from our proposed method with results from a few currently available methods that aim to characterize molecular evolution while also taking into account changes in amino acid properties, namely, the regression model in [10], TreeSAAP[25], and EvoRadical[9].

In [10], the first level of the model is the regression equation on $\left(y_{i,j}^{*}\right)$ as in equation (1), but it implicitly assumes independence among properties and independence among sites unlike our current model. The model in [10] is suitable for use when a few mostly independent amino acid properties are being analyzed whereas the new semiparametric model is better suited to the analysis of a large number of possibly correlated properties.

TreeSAAP uses the methods of [6] to classify nonsysnonymous substitutions into one of *M* categories, with higher numbered categories corresponding to sites showing radical changes and lower numbered categories used for sites showing conserved changes for a given property. For the analysis considered here, we used 8 categories where categories 6, 7, and 8 corresponded to sites showing radical changes, and categories 1 and 2 to sites showing conserved changes. Nonsynonymous changes are inferred from the ancestral reconstruction using the nucleotide substitution models in baseml implemented in PAML. We used a Bonferroni correction to correct for multiple comparisons.

EvoRadical implements the models of [9], which use partitions of amino acids to parameterize the rates of property-conserving and property-altering codon substitutions in a maximum likelihood framework. The model considers three types of substitutions: synonymous, property-conserving nonsynonymous and property-altering nonsynonymous which is a slight improvement from [8]. For analyses with multiple properties, one has to create different partitions for the different properties and run EvoRadical for each property.

### Posterior simulation

Various algorithms exist for posterior inference of DP mixtures - some of the most popular ones use (i) the Pólya urn characterization to marginalize out the unknown distribution(s) [26,27], (ii) a truncation approximation to the stick-breaking representation of the process which paves the way for the use of methods employed in finite mixture models [28,29], (iii) reversible jump MCMC or split-merge methods [30,31]. Some other recent approaches have also used variational methods [32] and slice samplers [33].

We use an extension of the finite mixture approximation discussed in [28] for its ease of implementation. Truncating *F* at a sufficiently large *K*, we write $\left(F^{\left(K\right)}=\overset{K}{\underset{k=1}{\sum }}\Pi_{k}\delta_{\theta_{k}^{*}}\right)$, with the weights *Π*_*k*_and locations $\left(\theta_{k}^{*}\right)$ generated as described earlier in this Section. Next we introduce configuration variables {*ζ*_*j*_} such that, for *k*=1,…,*K*, *ζ*_*j*_=*k* if and only if $\left(\theta_{j}=\theta_{k}^{*}\right)$. Similarly for *G*_*k*_, we truncate at a sufficient level *L*, and introduce another set of configuration variables {*ξ*_*i*,*k*_} where *ξ*_*i*,*k*_=*l*, with *l*=1,…,*L*, if and only if $\left(\theta_{i,k}^{*}=\varphi_{l,k}\right)$. Additional details about the algorithm are provided in the Appendix.

To determine the truncation levels *K* and *L*, we follow [29]. In particular, note that conditional on *ρ* (the DP concentration parameter), the tail probability $\left(\overset{\infty }{\underset{k=K}{\sum }}\Pi_{k}\right)$ has expectation {*ρ*/(1 + *ρ*)}^*K*−1^. Using prior guesses for *ρ*and acceptable tolerance levels for the tail probability to be small, one can then solve for the truncation level *K*. In our analyses, we used *K* and *L* in the range of 25 to 35. These values are in line with those used in other applications (for example, see [34]).

## Results and discussion

### Empirical exploration via simulation studies

We present two simulation studies to check the performance of the model under different scenarios. Additional simulation scenarios that may be of interest are available as an Additional file 1.

#### Simulation study 1

The setup for the first simulation is as follows. We generate values for the distinct regression coefficients (*ϕ*_*l*,*k*_) from a N(1,0.25). The number of distinct regression coefficients depends on the particular clustering structure for the corresponding simulation. Once we obtain the regression coefficients, we generate observations *y*_*i*,*j*_ from N(*ϕ*_*l*,*k*_*x*_*i*,*j*_,*σ*^2^=0.001). The *x*_*i*,*j*_s are obtained from the lysin data set described below with analyses for 32 properties, which implies *J*=32 and *I*=94.

We fitted the model in The Model subsection to the $\left(y_{i,j}^{*}\right)$s and $\left(x_{i,j}^{*}\right)$s, with the following modifications: (i) the NIRM is imposed on *β*_*i*,*j*_, so *φ*_*l*,*k*_=*ϕ*_*l*,*k*_ and (ii) *ϕ*_*l*,*k*_∼*G*_0_where *G*_0_∼N(*α*,*τ*^2^). We used *K*=25 and *L*=25 for the simulations. The MCMC algorithm was run with the following hyperpriors: *ρ*∼Ga(1,1), *γ*_*k*_∼Ga(1,1) for all *k*, *α*∼N(1,0.25). *σ*^2^∼Inv-Ga(100, 10) and *τ*^2^∼Inv-Ga(2,4) were chosen such that the prior means corresponded to the true values for these hyperparameters. Results are based on 15000 iterations, with the first 5000 discarded as burn-in. Convergence was assessed by running two chains where each chain was initialized by randomly assigning the *β*_*i*,*j*_s to different partitions. Posterior summaries based on the two chains were consistent with each other.

**Figure 2:**
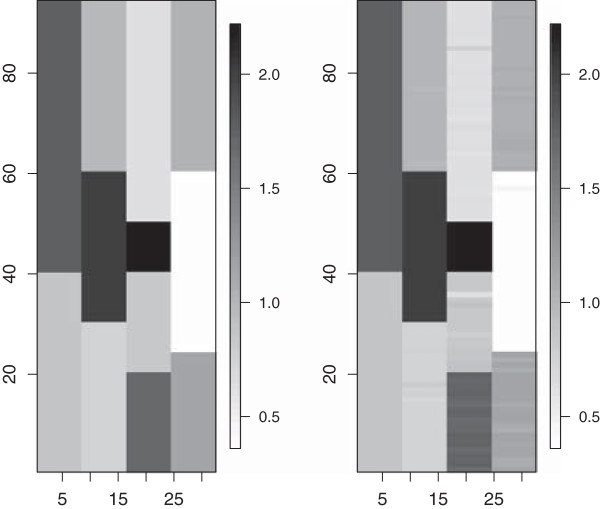
**Image plots for true *β***_**i**,*j*_**values (left panel) and posterior means **$\left({\hat{\beta }}_{i,j}\right)$**s (right panel).**

**Figure 3:**
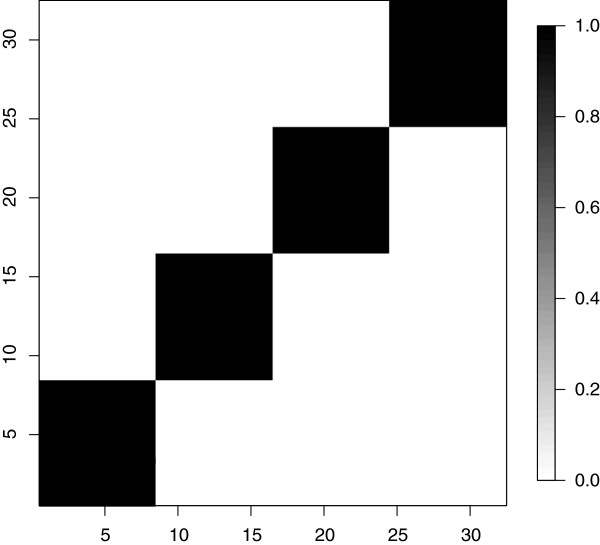
Marginal posterior probabilities of each pair of columns belonging to the same cluster.

In this scenario, we had four clusters for the columns, each with differing number of groups, leading to twelve distinct cluster combinations for the entire matrix of *β*_*i*,*j*_s (Figure 2, left panel). Figure 3 shows the marginal probabilities for any two columns (properties) of belonging to the same cluster. The model correctly identifies that there are 4 clusters for the columns and assigns each set of columns to its corresponding cluster with no uncertainty.

**Figure 4:**
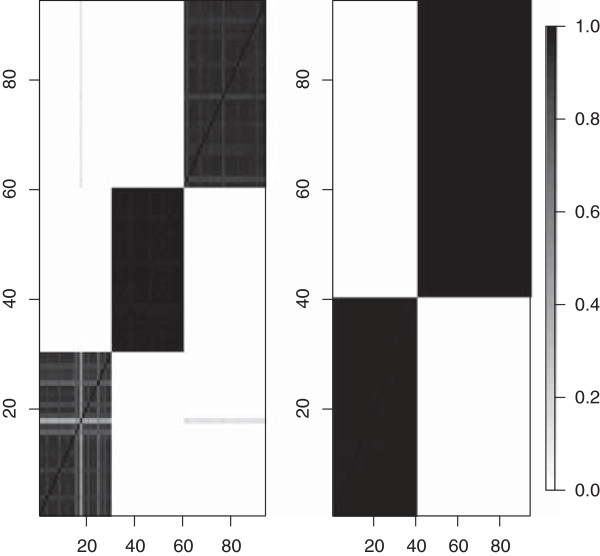
Marginal posterior probabilities of each pair of rows belonging to the same cluster for two different clusters of columns.

Similar graphical summaries obtained for the structure of rows within each cluster of columns show that the correct clustering structures for the rows, within each cluster of columns, are inferred (see Figure 4). For this level, however, there is some uncertainty about the membership of the clusters for a few rows. See, for example, the right panel of Figure 4. Some rows in cluster 1 (in the lower left) are sometimes being assigned to cluster 3 (top right). The distinct values of *ϕ* used for these two clusters were 0.73 and 0.98, therefore, it does not seem unreasonable to see some uncertainty in the assignment of clusters. Posterior means of $\left({\hat{\beta }}_{i,j}\right)$s agree closely with the true values as shown in Figure 2.

This scenario corresponds to the type of situation we expect on most real datasets: properties will cluster into groups and, within each group of properties, clusters of sites with similar responses can be clearly identified. Our results suggest that, as expected, the model is capable of identifying these multiple clusters with high accuracy and therefore accurately estimate the value of the regression coefficients. Other scenarios, including extreme cases where all properties belong to a common cluster while sites belong to one of several clusters, and cases where each property has a different effect on amino acid rates are available as Additional file 1.

To investigate the effect of the truncation levels and the priors on our model, we performed sensitivity analysis by varying the truncation levels as well as the different hyperparameters. Increasing the truncation level to 35 did not affect the results and the estimated posterior means of the *β*s showed close agreement with the true values. The analyses was also fairly robust to the choice of the priors, since varying the hyperparameters had almost no effect on the results. Decreasing the prior variance of *τ*^2^ makes the results marginally better, i.e., posterior means of the *β*_*i*,*j*_s, $\left({\hat{\beta }}_{i,j}\right)$s, are slightly closer to the true values.

#### Simulation study 2 - data simulated from a biological model

In our second simulation study the model is evaluated in the context of biological sequences generated from an evolutionary model. In particular, a Markov model was used to generate 20 sequences of 90 codons each. For the first one-third of the sites (sites 1-30) we used transition probabilities obtained from the codon-substitution model of [21] with equal equilibrium probabilities for all 61 codons. For the second one-third of the sites (sites 31-60), we modified the transition probability matrix from the previous step by increasing the probabilities of transitions between codons that have small distances for volume and decreasing the probabilities of transitions between codons that have large distances for volume - this was done to encourage only those changes that conserve volume in this part of the sequences. Finally, for the last one-third of the sites (sites 61-90), we modified the original transition probability to encourage radical changes in hydropathy. Thus, we increased some transition probabilities between codons that have very different hydropathy scores and decreased a few of those that have similar hydropathy scores. Note that, since the equilibrium probabilities are either uniform or roughly uniform across all sites, the correlation structure across properties is retained in the expected distances, which simplifies the interpretation of the results.

Once we obtained the sequences, we generated ancestral sequences using PAML, version 3.15, [20] and calculated observed and expected distances *y*_*i*,*j*_ and *x*_*i*,*j*_ for five properties, namely, hydropathy (*h*), volume (*M*_*v*_), polarity (*p*), isoelectric point (*pH*_*i*_) and partial specific volume (*V*^0^). Of these, *h* and *p* are correlated and so are *M*_*v*_and *V*^0^.

Our model was fitted with *K*=25 and *L*=25 as truncation levels. The prior distributions were the same as the ones used for our previous simulation. Results are based on 15000 iterations, of which the first 5000 were burn-in. There did not seem to be any obvious problems with convergence, which was assessed by visual inspection of trace plots of some of the parameters.

**Figure 5:**
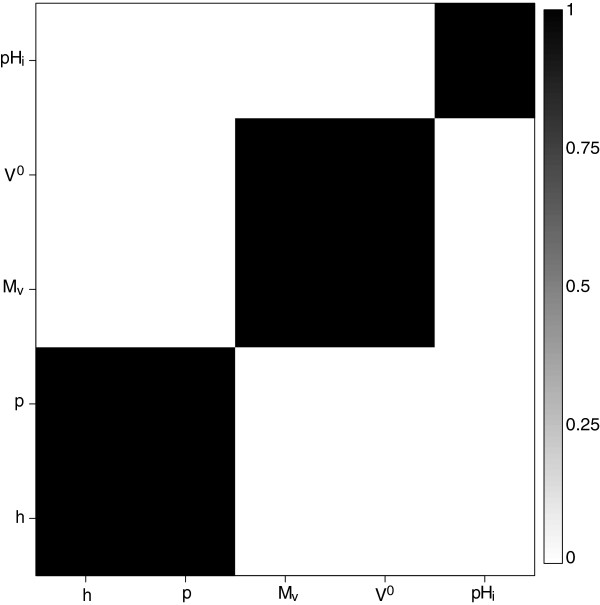
Marginal posterior probabilities of any two properties being in the same cluster for the data simulated under a biological model.

**Figure 6:**
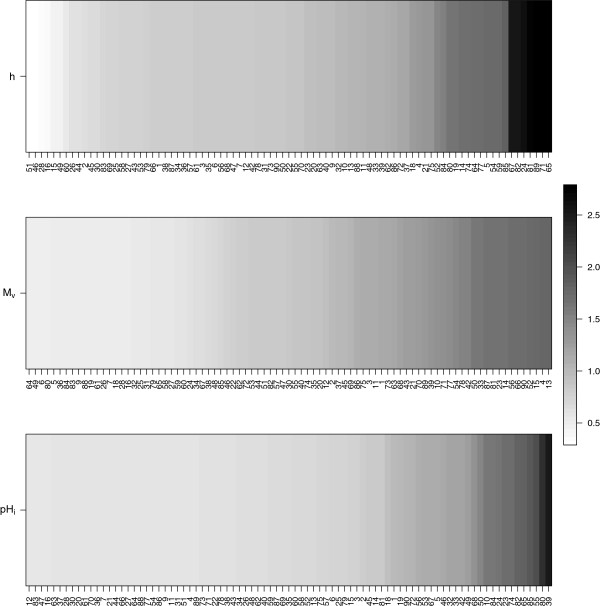
**Posterior means of *β***_**i,***j*_**s for the three clusters in Figure **5** for the simulated data under a biological model.** The sites are sorted according to the increasing value of posterior means.

**Figure 7:**
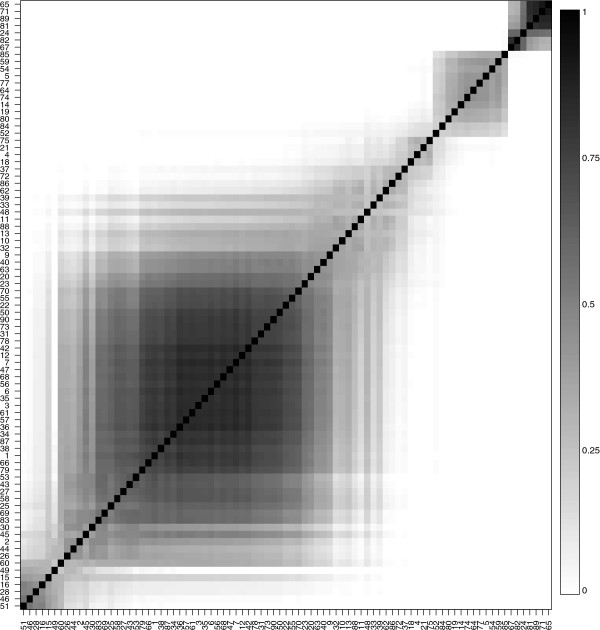
**Marginal posterior probabilities of any two sites for the simulated data being grouped together in the first cluster in Figure **5**.** The sites are sorted according to the increasing value of posterior means of *β*_*i*,*j*_s.

The analyses found that there were three clusters of properties - the first cluster has properties *h* and *p*, the second cluster comprised of properties *M*_*v*_ and *V*^0^ and the third cluster only had property *pH*_*i*_ as shown in Figure 5. Figure 6 shows the posterior means of *β*_*i*,*j*_s for representative properties of the three clusters in Figure 5. Sites 24, 65, 67, 71, 81, 82, and 89 have large posterior means $\left({\hat{\beta }}_{\mathrm{ij}}\right)$s for cluster 1 (*h* and *p*). These are also the same sites that show up in the small cluster at the top right in Figure 7. Specifically, Figure 7 shows how often any two sites in cluster 1 are grouped together. The sites in the lower left (16, 28, 46, 51) have small posterior means $\left({\hat{\beta }}_{i,j}\right)$s for these properties (*h* and *p*) and are grouped together more often. The big group of sites in the middle mostly seem to have mean $\left({\hat{\beta }}_{i,j}\right)$s around 1 while sites 81, 89, 71, and 65 have the largest $\left({\hat{\beta }}_{i,j}\right)$ values and very large probabilities of being clustered together in cluster 1. Thus, the model successfully identifies sites that have similar *β*_*i*,*j*_values in a specific cluster and groups them together. Groups of sites that change a property can also be identified for clusters 2 and 3 in Figure 5. In particular, for cluster 2 (*M*_*v*_ and *V*^0^), there is a big group of sites which conserve these properties. Most of these sites are in the central one-third portion (i.e., the portion that includes sites 31-60) which were simulated under a transition probability matrix that favors transitions that conserve volume. Finally, for cluster 3 (*pH*_*i*_) there is one large group of sites which conserve the property and one group comprising sites 39 and 80 which change the property greatly.

To better understand the performance of our method, we also analyzed the sequences generated above with the parametric regression model in [10], TreeSAAP[25], and EvoRadical[9]. Table 1 lists the thirty sites with the largest posterior means $\left({\hat{\beta }}_{i,j}\right)$s for *h*, and the thirty sites with the smallest posterior means $\left({\hat{\beta }}_{i,j}\right)$s for *M*_*v*_ for the regression model of [10] and also for our new semiparametric approach. Many of the same sites are identified by both methods, however, our new method performs slightly better than the regression model in [10]. In particular the new method identifies two additional sites in the 61-90 region as sites that change *h*.

**Table 1:** **Comparing results between models in [**[10]**] and the new semiparametric model, for the data simulated under a biological model**

	**Parametric regression **[10]	**Semiparametric regression**
30 sites with largest posterior mean $\left({\hat{\beta }}_{i,j}\right)$ for *h*	4, 5, 6, 10, 14, 18, 19, 21, 22, 23, 24, 33, 48, 52, 54, 59, **62**, **64**, **65**, **67**, **71**, **74**, **75**, **77**, **80**, **81**, **82**, **84**, **85**, **89**	4, 5, 14, 18, 19, 21, 24, 33, 37, 39, 48, 52, 54, 59, **62**, **64**, **65**, **67**, **71**, **72**, **74**, **75**, **77**, **80**, **81**, **82**, **84**, **85**, **86**, **89**
30 sites with lowest posterior mean $\left({\hat{\beta }}_{i,j}\right)$ for *M*_*v*_	5, 6, 7, 9, 16, 19, 24, 25, 26, 27, 28, **31**, **32**, **36**, **44**, **49**, **51**, **58**, **59**, **60**, 61, 64, 65, 67, 79, 80, 82, 83, 85, 88	5, 6, 7, 9, 16, 18, 19, 24, 25, 26, 27, 28, **31**, **32**, **34**, **36**, **38**, **49**, **58**, **59**, **60**, 61, 64, 65, 67, 79, 80, 83, 84, 88

Sites marked in bold are the ones which are in the region of interest - for *h* this is where radical changes were encouraged and for *M*_*v*_where small changes were encouraged while generating the sequences. Underlined sites are identified by both methods.

Table 2 lists sites that TreeSAAP finds significant for the different properties. All of the sites that TreeSAAP finds significant are also identified by our methods. However, note that once we correct for multiple comparisons in the TreeSAAP results, only one site (74) still remains significant. We note that the hierarchical specification of the priors in our models automatically accounts for multiple comparisons and no corrections are needed (see [10] for more discussion on this).

**Table 2:** Sites identified as significant by TreeSAAP for the different properties for the simulation study based on a biological model

**Property**	**Radically changing (1.645)**	**Radically changing (3.695)**	**Conserved (1.645)**	**Conserved (3.695)**
*h*	5, 59, **65**, **67**, **71**, **74**, **81**, **82**, **89**	**74**	36, 83	None
*p*	21, 24, 37, **64**, **65**, **67**, **71**, **74**, **75**, **81**, **82**, **89**	None	7, 18, 36, 49, 55	None
*M*_*v*_	10, 33, 66	None	5, 18, **36**, **49**	None
*V*^0^	10, 13, 33, 66	None	18, **36**	None
*pH*_*i*_	39, 55, 72	None	11, 64, 72	None

Values in parentheses denote the cut-off values for the *z*-test statistic. Sites marked in bold are in the region of interest.

Finally, we analyzed the sequences generated previously with EvoRadical using two different partitions [8] - one for *p* and the other for *M*_*v*_. We chose to run Evoradical with *p* instead of *h*, since a partition of the amino acids for polarity was already available in [8]. Additionally, given that *h* and *p* are correlated, we expect to see somewhat similar results for these two properties.

Table 3 lists site-specific results from EvoRadical. The sites listed have high posterior probabilities (>0.95) of being in the different site classes. This was the criterion that was used to identify significant sites in [9]. The results presented here correspond to Model A1 in [9] which uses *ω* for the nonsynonymous to synonymous substitution rate ratio for codons encoding amino acids with properties in the same partition, and *γ*measures the nonsynonymous to synonymous substitution rate ratio between codons for properties belonging to different partitions. While the sites listed for *p* somewhat match results from the other methods, the results for *M*_*v*_ are not in agreement. This is probably due to the fact that partitions are not always directly comparable with the amino acid distances. For example, under the volume partition of [8], both glycine and valine are small and glutamine is large, while looking at the volume scores glycine is very different from valine and glutamine. Thus, our models would consider a change from glycine to valine as radical, whereas for the partition-based method of [9], there would be no change. The fact that the user has to define a property-specific partition in advance, as opposed to directly working with the physicochemical distances, is one of the disadvantages of partition-based methods.

**Table 3:** **Sites that have high posterior probabilities (>0.95) of belonging to each site class for the different partitions for **EvoRadical** for the simulated data**

**Property**	***ω *≤1, ***γ ***≤1**	***ω *≤1, ***γ ***>1**	***ω *>1,VV ***γ ***≤1**	***ω *>1, ***γ ***>1**
*p*	None	None	None	1, 2, 5, 7, 10, 11, 12, 13, 14, 18, 19, 20, 26, 27, 30, 32, 33, 34, 36, 37, 42, 43, 47, 53, 57, 59, **61**, **62**, **63**, **64**, **66**, **67**, **68**, **69**, **72**, **73**, **74**, **75**, **77**, **82**, **83**, **86**, **87**, **88**, **90**
*M*_*v*_	None	None	None	2, 7, 9, 18, 19, 20, 22, 27, 31, 32, 36, 38, 53, 55, 61, 62, 64, 67, 72, 74, 86

Sites marked in bold are in the region of interest.

### Illustration with Lysin data

Our proposed model was applied to the sperm lysin data set which consisted of cDNA from 25 abalone species with 135 codons in each sequence [35]. Sites with alignment gaps were removed from all sequences, which resulted in 122 codons for the analysis presented here. The phylogeny of [35] and the codon substitution model M8 in PAML, version 3.15, [20] was used to generate the ancestral sequences. The model M8 uses a discretized beta distribution to model *ω* values between zero and one with probability *p*_0_ and allows for an additional positive selection category with *ω*>1 and probability *p*_1_.

The lysin data was analyzed with the model in The Model subsection with the 32 amino acid properties listed in Table 4. A few of the properties were chosen because of their functional importance. Some of the other properties have been previously used in analyses by [25]. Only sites which showed at least one nonsynonymous change were retained for the final analysis, which led to a data set with 94 sites. We used *K*=25 and *L*=35 as truncation levels for this data. The prior distributions with the following hyperparameters were used in the analysis. The DP concentration parameters *ρ* and *γ*_*k*_ were assumed to follow a Ga(1,1). *λ*, the prior probability for *ϕ*_*l*,*k*_ being 0, was assumed to follow a Beta(2,8) which implied that about 20% of the unique *β*_*i*,*j*_s were expected to be 0 *a priori*. *a*_*κ*_and *b*_*κ*_, the hyperparameters for the prior of $\left(\vartheta_{l,k}^{2}\right)$ when *ϕ*_*l*,*k*_is 0, were chosen as 2 and 100 which implied a prior mean of 0.01. When *ϕ*_*l*,*k*_ is different from zero, $\left(a_{\sigma }^{*}=2\right)$ and $\left(b_{\sigma }^{*}=10\right)$ control the prior for $\left(\vartheta_{l,k}^{2}\right)$. *V*_0_, the scale factor for $\left(\vartheta_{l,k}^{2}\right)$, was fixed at the ratio of prior means of *σ*^2^and $\left(\tau_{i}^{2}\right)$ (the variance terms in the regression model in [10] for which we had used prior means of 0.1 and 0.01 respectively). Finally, the *α*_*k*_s were assumed to follow a N(1, 0.25) to conform to our prior assumption of neutrality for the properties. Results are based on 20000 iterations, of which the first 10000 were burn-in. Convergence was assessed by visual inspection of trace plots of some of the parameters and there did not seem to be any obvious problems with convergence.

**Table 4:** List of 32 amino acid properties used in the analysis

**AAindex accession number (if available)**	**Property**	**Symbol**	**AAindex accession number (if available)**	**Property**	**Symbol**
KYTJ820101	Hydropathy	*h*	∗	Helical contact area	*C*_*a*_
GRAR740103	Molecular volume	*M*_*v*_	ZIMJ680104	Isoelectric point	*pH*_*i*_
MANP780101	Surrounding hydrophobicity	*H*_*p*_	OOBM770103	Long-range non-bonded energy	*E*_*l*_
ZIMJ680103	Polarity(Zimmerman)	*p*_*zim*_	∗	Mean r.m.s. fluctuation displacement	*F*
CHOP780201	Alpha-helical tendencies	*P*_*α*_	FASG760101	Molecular weight	*M*_*w*_
GRAR740102	Polarity(Grantham)	*p*	∗	Normalized consensus hydrophobicity	*H*_*nc*_
PONP800108	Average number of surrounding residues	*N*_*s*_	COHE430101	Partial specific volume	*V*^0^
∗	Power to be at the C-terminal	*α*_*c*_	WOEC730101	Polar requirement	*P*_*r*_
GRAR740101	Composition	*c*	∗	Power to be at the middle of alpha-helix	*α*_*m*_
∗	Compressibility	*K*^0^	∗	Power to be at the N-terminal	*α*_*n*_
FAUJ880113	Equilibrium constant (ionization of COOH)	*pK*^*′*^	MCMT640101	Refractive index	*μ*
CHOP780202	Beta-structure tendencies	*P*_*β*_	OOBM770102	Short and medium range non-bonded energy	*E*_*sm*_
ZIMJ680102	Bulkiness	*B*_*l*_	PONP800107	Solvent accessible reduction ratio	*R*_*a*_
∗	Buriedness	*B*_*r*_	∗	Thermodynamic transfer hydrophobicity	*H*_*t*_
∗	Chromatographic index	*R*_*F*_	OOBM770101	Total non-bonded energy	*E*_*t*_
CHAM830101	Coil tendencies	*P*_*c*_	CHOP780101	Turn tendencies	*P*

Properties marked by ∗ are from [36].

**Figure 8:**
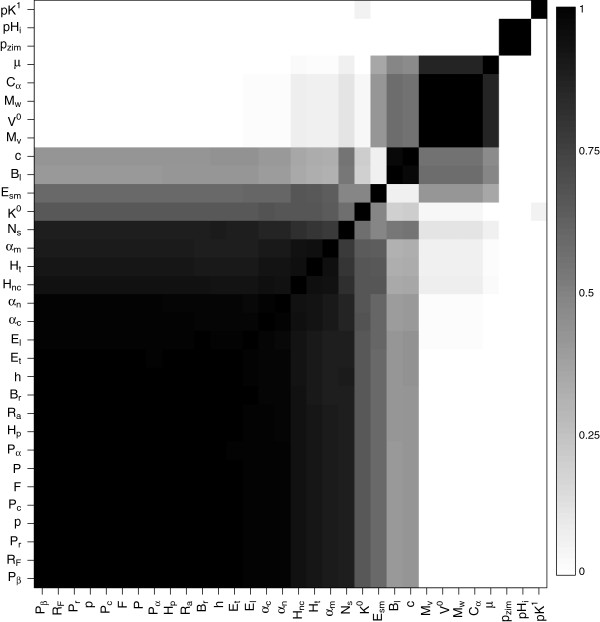
Marginal posterior probabilities of any two properties being in the same cluster for the lysin data.

Figure 8 shows the marginal posterior probabilities of any two properties being assigned to the same cluster. There seem to be four mostly distinct clusters in the properties in our list. The biggest cluster consists of 20 properties that are related to polarity and hydropathy. All 20 properties are assigned to this cluster with very high probability. The next cluster is comprised of the properties *B*_*l*_, and *c*. There is also a fairly big cluster whose members are related to volume (*M*_*v*_,*V*^0^,*M*_*w*_,*C*_*α*_,*μ*). *p*_*zim*_, which is correlated with *p* to some extent, is clustered with *pH*_*i*_with which it shows a large correlation value (about 0.9). There is some uncertainty regarding the membership of *K*^0^ and *E*_*sm*_, since both of them are assigned to the largest cluster about 50% of the time, while *E*_*sm*_ is clustered with properties related to volume to a lesser extent. *pK*^1^is the only property that is almost never clustered with other properties.

**Figure 9:**
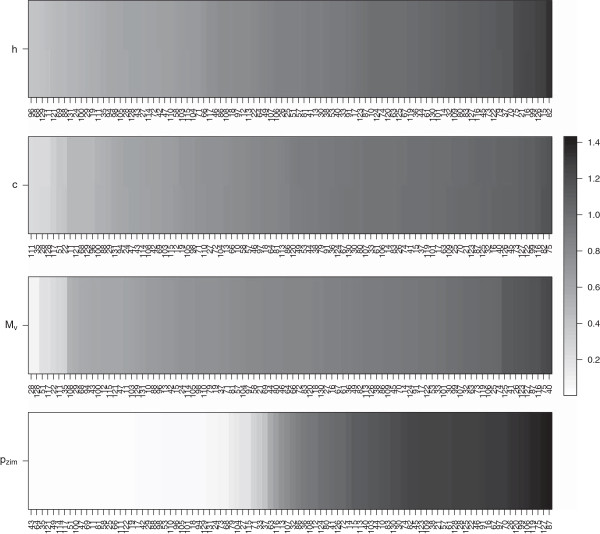
**Posterior means **$\left({\hat{\beta }}_{i,j}\right)$**s for the four clusters (denoted by representative properties) in Figure **8** for lysin.** The sites are sorted according to the increasing value of posterior means.

Site specific results based on the posterior means (denoted by $\left({\hat{\beta }}_{i,j}\right)$s), for one representative property each from the four clusters in Figure 8 are shown in Figure 9. The sites are sorted according to the increasing value of mean $\left({\hat{\beta }}_{i,j}\right)$ for each image. Sites on the far right radically change properties in each group. For example, most of the sites that appear on the far right, like sites 15, 16, 21, 75, 82, 99 and 126, for cluster 1 (represented by *h*) have $\left({\hat{\beta }}_{i,j}\right)$ values of 1.2-1.4. There seem to be more sites radically changing properties in cluster 1 than in clusters 2 (represented by *c*) or 3 (represented by *M*_*v*_). The first three clusters also have a fairly large number of sites with mean $\left({\hat{\beta }}_{i,j}\right)$ between 0 and 1. This is different from what we see for cluster 4 (represented by *p*_*zim*_), which corresponds to properties *p*_*zim*_and *pH*_*i*_. A large number of sites in cluster 4 strongly conserve the properties (e.g., sites 35, 43, 49, 51, 64, 114, 117, 121), as is evident by the very small mean $\left({\hat{\beta }}_{i,j}\right)$s for sites in the far left, unlike in the other clusters.

**Figure 10:**
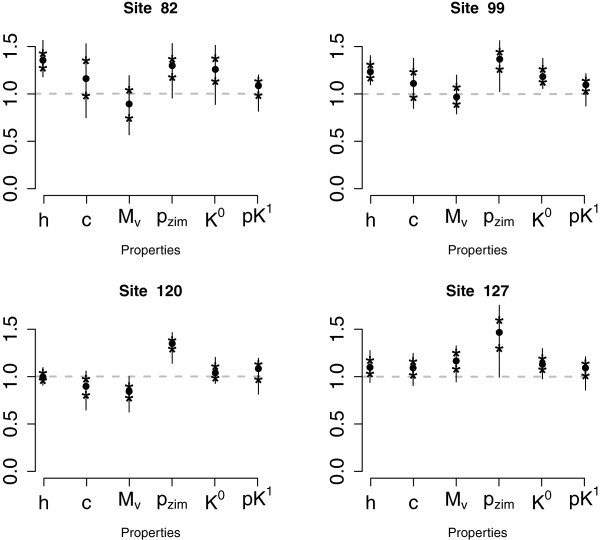
**Posterior summaries of *β***_***i,j***_**s different from zero for sites 82, 99, 120 and 127 in lysin data.** The first 4 properties on the x-axis belong to 4 different clusters and the next 2 do not belong to any specific cluster all the time. The vertical lines are 90% posterior intervals of the *β*_*i*,*j*_s that are different from 0, the medians (filled circles) and the 25^*th*^ and 75^*th*^ percentiles (stars) are highlighted.

Figure 10 shows the posterior summaries of *β*_*i*,*j*_s different from zero for sites 82, 99, 120 and 127 for properties belonging to different clusters. Of these, sites 120 and 127 were found to be under positive selection by PAML, while sites 82, 99 and 127 were identified as radically changing some of the properties by the regression model in [10]. The sites show different behavior for the different properties, for example, site 82 shows radical changes for *h*, while it conserves *M*_*v*_. We can also see similarities in the posterior summaries across sites. For example, for property *pK*^1^ sites 82, 120 and 127 have similar values for *β*_*i*,*j*_. One of the advantages of using the semiparametric approach is that we can identify groups of sites that either conserve or radically change a set of similar amino acid properties. For example, sites 122 and 127 both seem to be altering the amino acid properties in the first large cluster of properties related to *p* and *h*. However, sites 122 and 127 have a very different behavior in cluster 4 related to *p*_*zim*_: site 122 strongly conserves properties in this cluster while site 127 radically changes them.

Table 5 lists sites that are highly conserved with posterior mean $\left({\hat{\beta }}_{i,j}\right)$s less than 0.4 for the different clusters. The largest number of highly conserved sites appears in cluster 4, which includes properties *p*_*zim*_and *pH*_*i*_, in agreement with Figure 9. Some of these sites like 35, 51, 111 and 117 also conserve properties in clusters 2 and 3. A number of them, such as sites 28, 35, 58, 66, 94, 104, 117, and 128 are also identified as sites under negative selection by methods that take into account the relative rate of nonsynonymous to synonymous rate ratio, such as those implemented in PAML[20]. In order to determine which sites are under positive and negative selection by PAML, we follow an approach similar to that used by [35] in the analysis of the lysin data. In particular, [35] found that PAML model M8, which supports positive selection, is the model that better fits the lysin data. Therefore, we classified sites as negatively selected if the estimated *ω* was smaller than 0.3 and if Pr(*ω*>1|data)<0.5 using PAML model M8. Results comparing sites conserving or radically changing a small group of properties with sites inferred to be under positive or negative selection by PAML was also presented in [10].

**Table 5:** **Strongly conserved sites (**$\left({\hat{\beta }}_{\mathrm{ij}}<0.4\right)$**) for lysin data for different clusters**

**Cluster**	**Site number**
1	96
2 and 3	22, 28, 35, 51, 111, 117, 128
	11, 17, 18, 19, 24, 25, 27, 29, 33, 35, 42, 43, 47, 49, 51,
4	53, 58, 64, 66, 68, 69, 71, 73, 79, 81, 88, 94, 96, 98, 100,
	101, 104, 105, 110, 111, 114, 115, 117, 121, 122, 129, 131

The results are fairly robust to the choice of different hyperparameter values. Note that the scale factor for $\left(\vartheta_{l,k}^{2}\right)$ ultimately affects the variation in the *β*_*i*,*j*_values, and it is advisable to choose it so that the prior variance for the unique *β*_*i*,*j*_s is not too large.

## Conclusions

In this paper, we present a Bayesian hierarchical regression model with a nested infinite relational model on the regression coefficients. The model is capable of identifying sites which show radical or conserved amino acid changes. The (almost sure) discreteness of the DP realizations induces clustering at the level of properties which is analogous to the factor model in [11], with the advantage being that the nonparametric method automatically determines the appropriate number of clusters. The multi-level clustering ability of the NIRM also induces clustering at the level of sites and allows us to capture skewness and heterogeneity in the distribution of the random effects distribution associated with each cluster of properties.

The main advantage of the models we have described is their ability to simultaneously handle multiple properties with potentially correlated effects on molecular evolution. Our simulations suggest that our models are flexible but robust, being capable of dealing with a range of situations including those where properties are perfectly correlated, as well as those where all properties are uncorrelated. Our semiparametric regression models also work well, particularly in comparison with the regression model in [10], TreeSAAP and EvoRadical, when applied to DNA sequence data generated from an evolutionary model. In addition, the analysis of the lysin data suggests that the model leads to reasonable results.

The NIRM that is the basis of our model defines a separately exchangeable prior on matrices. This means that the prior is invariant to the order in which properties and sites are included. This is due to the fact that the rows as well as the columns of the parameter of interest are independent draws from a DP. From the point of view of modeling multiple properties, this is a highly desirable property. However, assuming that DNA sites are exchangeable can be questionable. Although this is a potential limitation of our model, we should note that the assumption of independence across sites (which is a stronger assumption than exchangeability) underlies all the methods discussed in the Background section. If information about the 3-dimensional structure of the encoded protein or other sequence specific information that can guide the construction of the dependence model is available, our model could be easily extended to account for this feature. In the absence of such information, exchangeability across DNA sites seems to be a reasonable prior assumption. Indeed, in contrast to the most common independence assumption, our exchangeability assumption allows us to explain correlations at the level of sites.

In our applications, we have used codon substitution models for reconstructing ancestral sequences as we wished to compare our methods to other methods for detecting selective sites that also use codon substitution models, such as those implemented in PAML and EvoRadical. However, it is possible to perform the proposed Bayesian semiparametric analyses using amino acid substitution models instead of codon substitution models. Note that the substitution model is only used in the calculation of the observed distances. First, we infer the ancestral sequences under a specific substitution model and a given phylogeny. We then compute the observed distances for a given property and a given site as the mean absolute difference in property scores due to all nonsynonymous substitutions at that site, where the nonsynonymous substitutions are counted by comparing the DNA sequences between two neighboring nodes in the phylogeny. The reconstructed ancestral sequences, and therefore the observed distances in our model, may differ under different substitution models, but the method can be implemented under any substitution model, including amino acid substitution models. The gain in execution time from using amino acid substitution models instead of codon-based ones could potentially be significant if the uncertainty in the alignment/phylogeny/ancestral level is taken into account.

Finally, it is important to note that the “observed” distances are not really directly observed, but are instead constructed from ancestral sequences and, therefore, subject to error. A simple way to account for this additional level of uncertainty is to modify the computation of expected distances by incorporating the ideas of [37]. This approach was previously employed in [10], with little impact on the final results.

## Appendix: details about the Gibbs sampler

The truncations and the introduction of the configuration variables imply that (2) and (3) can be written as

(5) $$\zeta_{j}|\{\Pi_{k}\}∼\overset{K}{\underset{k=1}{\sum }}\Pi_{k}\delta_{\theta_{k}^{*}}\;\xi_{i,k}|\{w_{l,k}\}∼\overset{L}{\underset{l=1}{\sum }}w_{l,k}\delta_{\varphi_{l,k}}$$

with *φ*_*l*,*k*_∼*G*_0*lk*_and *Π*_*k*_ and *w*_*l*,*k*_ being the appropriate stick breaking weights. Writing the model as in (5) helps in obtaining the forms of the full conditionals as below.

The column indicators *ζ*_*j*_ for *j*=1,…,*J* are sampled from a multinomial distribution with probabilities

$$P(\zeta_{j}=k|\cdots \;)=q_{j}^{k}\propto \overset{L}{\underset{l=1}{\sum }}\underset{\left\{i:\xi_{i,k}=l\right\}}{\prod }\Pi_{k}\text{N}(y_{i,j}^{*}|\phi_{l,k}x_{i,j}^{*},\vartheta_{l,k}^{2}),$$

where $\left(\vartheta_{l,k}^{2}\right)$ is $\left(\vartheta_{l,k}^{2}\right)$ if *ϕ*_*l*,*k*_=0 or is $\left(\vartheta_{l,k}^{2}/n_{i}^{O}\right)$ if *ϕ*_*l*,*k*_ is different from zero. *Π*_*k*_ is sampled in two parts: first, by generating *v*_*k*_from a $\left(\text{Beta}(1+m_{k},\rho +\overset{K}{\underset{s=k+1}{\sum }}m_{s})\right)$ for *k*=1,…,*K*−1 and *v*_*K*_=1, where *m*_*k*_is the number of columns assigned to cluster *k* and then, by constructing $\left(\Pi_{k}=v_{k}\overset{k-1}{\underset{s=1}{\prod }}(1-v_{s})\right)$.

For *i*=1,…,*I*and *k*=1,…,*K*, the indicators *ξ*_*i*,*k*_are also sampled from a multinomial with probabilities of the form

$$P(\xi_{i,k}=l|\cdots \;)=p_{i,k}^{l}\propto \underset{\left\{j:\zeta_{j}=k\right\}}{\prod }w_{l,k}\text{N}(y_{i,j}^{*}|\phi_{l,k}x_{i,j}^{*},\vartheta_{l,k}^{2}).$$

The updated weights *w*_*l*,*k*_ are sampled in a manner similar to the *Π*_*k*_, i.e., *u*_*l*,*k*_ are generated from a $\left(\text{Beta}(1+n_{l,k},\gamma_{k}+\overset{L}{\underset{r=l+1}{\sum }}n_{\mathrm{lr}})\right)$ for *l*=1,…,*L*−1 and *u*_*Lk*_=1, where *n*_*l*,*k*_ is the number of *β*_*i*,*j*_s assigned to atom *l* of cluster *k* and then, by constructing $\left(w_{l,k}=u_{l,k}\overset{l-1}{\underset{r=1}{\prod }}(1-u_{r,k})\right)$.

Following [18], the DP concentration parameters *ρ*and *γ*_*k*_ are sampled in two steps by introducing auxiliary variables *η*_1_and *η*_2_. First, sample *η*_1_from

$$p(\eta_{1}|\rho ,\cdots \;)=\mathrm{Beta}(\rho +1,J)$$

and then *ρ*from

$$\begin{array}{l} p(\rho |\eta_{1},\cdots \;)=\frac{a_{\rho }+n_{\zeta }^{*}-1}{a_{\rho }+n_{\zeta }^{*}-1+J(b_{\rho }-\text{log}(\eta_{1}))}\\ \;\times \text{Ga}(a_{\rho }+n_{\zeta }^{*},b_{\rho }-\text{log}(\eta_{1}))\\ \;+\frac{J(b_{\rho }-\text{log}(\eta_{1}))}{a_{\rho }+n_{\zeta }^{*}-1+J(b_{\rho }-\text{log}(\eta_{1}))}\\ \;\times \text{Ga}(a_{\rho }+n_{\zeta }^{*}-1,b_{\rho }-\text{log}(\eta_{1})), \end{array}$$

where $\left(n_{\zeta }^{*}\right)$ is the number of unique column indicators *ζ*_*j*_. Similarly, for each *k*=1,…,*K*,

$$p(\eta_{2}|\gamma_{k},\cdots \;)=\text{Beta}(\gamma_{k}+1,I),$$

$$\begin{array}{l} p(\gamma_{k}|\eta_{2},\cdots \;)=\frac{a_{\gamma }+m_{\xi ,k}^{*}-1}{a_{\rho }+m_{\xi ,k}^{*}-1+I(b_{\gamma }-\text{log}(\eta_{2}))}\\ \;\times \text{Ga}(a_{\gamma }+m_{\xi ,k}^{*},b_{\gamma }-\text{log}(\eta_{2}))\\ \;+\frac{I(b_{\gamma }-\text{log}(\eta_{2}))}{a_{\gamma }+m_{\xi ,k}^{*}-1+I(b_{\gamma }-\text{log}(\eta_{2}))}\\ \;\times \text{Ga}(a_{\gamma }+m_{\xi ,k}^{*}-1,b_{\gamma }-\text{log}(\eta_{2})), \end{array}$$

where $\left(m_{\xi ,k}^{*}\right)$ is the number of unique row indicators *ξ*_*i*,*k*_, for a specific cluster of columns *k*.

To sample the unique $\left(\varphi_{l,k}=(\phi_{l,k},\vartheta_{l,k}^{2})\right)$s given in (4), we introduce a set of indicator variables *ψ*_*l*,*k*_which take the value 1 when *ϕ*_*l*,*k*_is different from zero. For *l*=1,…,*L*and *k*=1,…,*K*, *ψ*_*l*,*k*_, $\left(\vartheta_{l,k}^{2}\right)$ and *ϕ*_*l*,*k*_ are jointly sampled in the following way - *ψ*_*l*,*k*_ is sampled by integrating out *ϕ*_*l*,*k*_and $\left(\vartheta_{l,k}^{2}\right)$ from its full conditional, $\left(\vartheta_{l,k}^{2}\right)$ is sampled conditional on *ψ*_*l*,*k*_ and *ϕ*_*l*,*k*_ is sampled conditional on both the corresponding *ψ*_*l*,*k*_and $\left(\vartheta_{l,k}^{2}\right)$, i.e.,

$$\begin{array}{cc} p(\psi_{l,k},\vartheta_{l,k}^{2},\phi_{l,k}|\cdots \;)\;=\; & p(\psi_{l,k}|\cdots \;)p(\vartheta_{l,k}^{2}|\psi_{l,k},\cdots \;)\\ \;\times p(\phi_{l,k}|\psi_{l,k},\vartheta_{l,k}^{2},\cdots \;) \end{array}$$

with the individual expressions obtained as follows.

First, let $\left(\Omega_{l,k}^{i,j}=\{(i,j):\xi_{i\zeta_{j}}=l,\zeta_{j}=k\}\right)$. Then,

$$\begin{array}{l} p(\psi_{l,k}|\cdots \;)\propto \lambda \int \left[\underset{\overset{i,j}{\underset{l,k}{\Omega }}}{\prod }\text{N}(\overset{*}{\underset{i,j}{y}}|0,\overset{2}{\underset{l,k}{\vartheta }})\right]\text{IG}(\overset{2}{\underset{l,k}{\vartheta }}|a_{\kappa },b_{\kappa })\text{d}(\vartheta_{l,k}^{2})\\ \;+(1-\lambda )\int \int \left[\underset{\overset{i,j}{\underset{l,k}{\Omega }}}{\prod }\text{N}(\overset{*}{\underset{i,j}{y}}|\phi_{l,k}\overset{*}{\underset{i,j}{x}},\overset{2}{\underset{l,k}{\vartheta }}/\overset{O}{\underset{i}{n}})\right]\\ \;\times \text{N}(\phi_{l,k}|\alpha_{k},\vartheta_{l,k}^{2}/V_{0})\text{IG}(\vartheta_{l,k}^{2}|a_{\sigma }^{*},b_{\sigma }^{*})\text{d}(\phi_{l,k})\text{d}(\vartheta_{l,k}^{2}). \end{array}$$

$$p(\vartheta_{l,k}^{2}|\psi_{l,k},\cdots \;)=\left\{\begin{array}{c} \text{IG}\left(\frac{I^{*}J^{*}}{2}+a_{\kappa },{\left[\frac{1}{b_{\kappa }}+\sigma_{1,\text{scale}}\right]}^{-1}\right)\;\text{if}\;\;\psi_{l,k}=0\\ \text{IG}\left(\frac{I^{*}J^{*}}{2}+a_{\sigma }^{*},{\left[\frac{1}{b_{\sigma }^{*}}+\sigma_{2,\mathrm{scale}}\right]}^{-1}\right)\;\text{if}\;\psi_{l,k}=1, \end{array}\right)$$

where $\left(I^{*}J^{*}=\underset{i,j}{\sum }1_{\left\{\xi_{i\zeta_{j}}=l,\zeta_{j}=k\right\}}\right)$ and the update terms are given by $\left(\sigma_{1,\mathrm{scale}}=\underset{\Omega_{l,k}^{i,j}}{\sum }\frac{y_{i,j}^{*2}}{2}\right)$ and $\left(\sigma_{2,\mathrm{scale}}=\frac{\alpha_{k}^{2}V_{0}}{2}+\underset{\Omega_{l,k}^{i,j}}{\sum }\frac{n_{i}^{O}y_{i,j}^{*2}}{2}-\frac{{\left(\alpha_{k}V_{0}+\underset{\Omega_{l,k}^{i,j}}{\sum }n_{i}^{O}y_{i,j}^{*}x_{i,j}^{*}\right)}^{2}}{2(V_{0}+\underset{\Omega_{l,k}^{i,j}}{\sum }n_{i}^{O}x_{i,j}^{*2})}\right)$.

$$p(\phi_{l,k}|\psi_{l,k},\vartheta_{l,k}^{2},\cdots \;)=\left\{\begin{array}{c} 0\;\text{if}\;\;\psi_{l,k}=0\\ \text{N}(m_{\phi },C_{\phi })\;\;\text{if}\;\;\psi_{l,k}=1, \end{array}\right)$$

where $\left(m_{\phi }=\left(\frac{\alpha_{k}V_{0}+\underset{\Omega_{l,k}^{i,j}}{\sum }n_{i}^{O}y_{i,j}^{*}x_{i,j}^{*}}{V_{0}+\underset{\Omega_{l,k}^{i,j}}{\sum }n_{i}^{O}x_{i,j}^{*2}}\right)\right)$ and $\left(C_{\phi }=\frac{\vartheta_{l,k}^{2}}{V_{0}+\underset{\Omega_{l,k}^{i,j}}{\sum }n_{i}^{O}x_{i,j}^{*2}}\right)$.

The full conditional of *λ* is given by

$$p(\lambda |\cdots \;)∼\text{Beta}(a_{\lambda }+\underset{l,k}{\sum }1_{\left\{\psi_{l,k}=0\right\}},b_{\lambda }+\underset{l,k}{\sum }1_{\left\{\psi_{l,k}=1\right\}}).$$

Finally, for *k*=1,…,*K*, the full conditional of *α*_*k*_is given by

$$p(\alpha_{k}|\cdots \;)∼\text{N}(m_{\alpha }^{*},C_{\alpha }^{*})$$

where

$$C_{\alpha }^{*}=\frac{1}{\left(\frac{1}{C_{\alpha }}+\underset{\left\{l:\psi_{l,k}=1\right\}}{\sum }\frac{V_{0}}{\vartheta_{l,k}^{2}}\right)}$$

and

$$m_{\alpha }^{*}=C_{\alpha }^{*}\left(\frac{m_{\alpha }}{C_{\alpha }}+\underset{\left\{l:\psi_{l,k}=1\right\}}{\sum }\frac{V_{0}\phi_{l,k}}{\vartheta_{l,k}^{2}}\right)$$

### Software availability

The R code implementing the models in the paper is freely available at http://www.ams.ucsc.edu/~raquel/software/.

## Competing interests

The authors declare that they have no competing interests.

## Authors’ contributions

SD, AR and RP formulated the model. SD performed the analyses and drafted the manuscript. AR and RP revised the manuscript draft. All authors read and approve the final version of the manuscript.

## Supplementary Material

Additional file 1 Additional simulations are provided in a separate supplemental file. Click here for file
