# Supplementary material for: Bayesian semiparametric regression models to characterize molecular evolution
Source: BMC Bioinformatics. 2012 Oct 30;13:278. doi: 10.1186/1471-2105-13-278 (PMC3577475; doi:10.1186/1471-2105-13-278)
Supplement: Additional file 1 — Additional simulations are provided in a separate supplemental file. [file 1471-2105-13-278-S1.pdf]

Online Supplement for Bayesian Semiparametric Regression Models to Characterize Molecular Evolution

Saheli Datta saheli@ams.ucsc.edu
Abel Rodriguez abel@ams.ucsc.edu
Raquel Prado raquel@ams.ucsc.edu

Additional Simulations

The setup for the simulations are as follows. We generate values for the distinct regression coefficients ($\phi_{l,k}$) from a $\mathsf{N}(1, 0.25)$. The number of distinct regression coefficients depends on the particular clustering structure for the corresponding simulation. Once we obtain the regression coefficients, we generate observations $y_{i,j}$ from $\mathsf{N}(\phi_{l,k}x_{i,j}, \sigma^2 = 0.001)$. The $x_{i,j}$s are obtained from the lysin data set described below with analyses for 32 properties, which implies $J = 32$ and $I = 94$, unless otherwise mentioned.

We fitted the model in Section 2 of the main paper to the $y^*_{i,j}$s and $x^*_{i,j}$s, with the following modifications: (i) the NIRM is imposed on $\beta_{i,j}$, so $\varphi_{l,k} = \phi_{l,k}$ and (ii) $\phi_{l,k} \sim G_0$ where $G_0 \sim \mathsf{N}(\alpha, \tau^2)$. We used $K = 25$ and $L = 25$ for the simulations. The MCMC algorithm was run with the following hyperpriors: $\rho \sim \mathsf{Ga}(1, 1)$, $\gamma_k \sim \mathsf{Ga}(1, 1)$ for all k, $\alpha \sim \mathsf{N}(1, 0.25)$. $\sigma^2 \sim \mathsf{Inv\text{-}Ga}(100, 10)$ and $\tau^2 \sim \mathsf{Inv\text{-}Ga}(2, 4)$ were chosen such that the prior means corresponded to the true values for these hyperparameters. For all the simulations, results are based on 15000 iterations, of which the first 5000 are burn-in. Convergence was assessed by running two chains where each chain was initialized by randomly assigning the $\beta_{i,j}$s to different partitions. Posterior summaries based on the two chains were consistent with each other.

Simulation Study 3

For this simulation, all the columns were assumed to belong to the same cluster. Six distinct $\phi_{l,k}$ values were used to generate the observations $y_{i,j}$ from the appropriate Normal density. Posterior summaries of the column cluster indicators revealed that the analysis indeed concludes all the columns belong to the same cluster. Clustering within columns was also inferred correctly. There was no uncertainty associated with the cluster memberships at either level. In this case also, posterior means of the $\beta_{i,j}$s showed very close agreement with true values of $\phi_{l,k}$ used to generate the data, as shown in Figure 1.

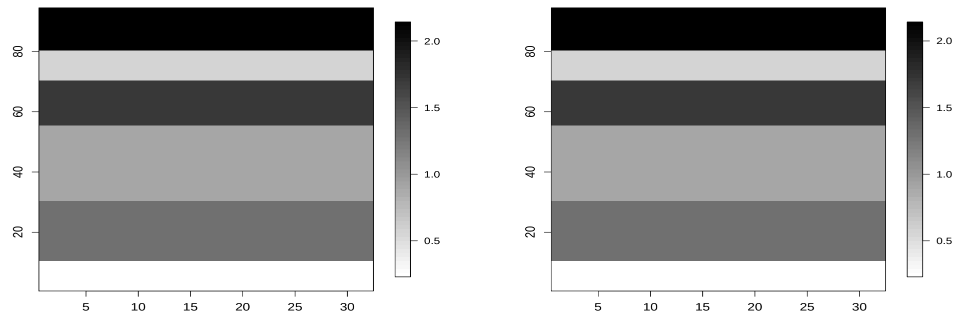

Figure 1: Image plots for true $\beta_{i,j}$ values (left panel) and posterior means $\hat{\beta}_{i,j}$s (right panel).

Simulation Study 4

For this simulation we chose a scenario where each column was different from the other. The number of columns for this simulation was 10 (so, $J = 10$). The number of rows in this simulation was 30. The first 18 rows were assumed to have the same $\phi_{l,k}$ for all the columns while the remaining $\phi_{l,k}$s were generated independently from $\mathsf{N}(1, 0.25)$.

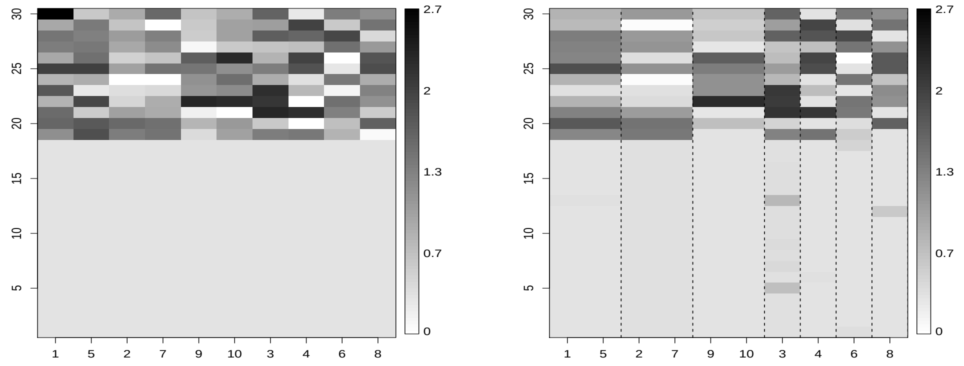

Figure 2: Image plots for true $\beta_{i,j}$ values (left panel) and posterior means of the $\beta_{i,j}$s (right panel). The columns have been arranged according to the clustering inferred by the model.

In this case, the model infers that there are 7 clusters for the columns, with very little uncertainty about the cluster memberships. For each cluster of column, the model assigned the first 18 rows to the same cluster with fairly high probability (> 0.83). In case of the remaining 12 rows, rows were assigned to the same cluster if the corresponding true ϕs were close. Figure 2 shows the true $\beta_{i,j}$ values and the estimated posterior means. While the images seem reasonably close, small differences do exist. For example, in Figure 2 (right panel) since columns 1 and 5 were assigned to the same cluster with very high probability,

we have $\mathsf{E}\{\beta_{30,1}|\text{Data}\} = \mathsf{E}\{\beta_{30,5}|\text{Data}\} = 0.8$, while the true values were $\beta_{30,1} = 2.65$ and $\beta_{30,5} = 0.56$ respectively. In spite of these differences, for each of the columns that were clustered together more than 2/3 of the true $\beta_{i,j}$s were very close (less than 0.1 difference).

Simulation Study 5

Our final simulation study was designed to investigate the extreme case where the rows and the columns were all generated independently. We considered 10 columns and 30 rows for this scenario. All 300 $\beta_{i,j}$s were generated independently from $\mathsf{N}(1, 0.25)$.

The model correctly infers that all 10 columns are independent. In cases where the true $\beta_{i,j}$ values are close for different i for a fixed j, a few of the rows are sometimes clustered together. As in the previous simulations, the posterior means of the $\beta_{i,j}$s are good estimates of the true $\beta_{i,j}$s as evident from Figure 3.

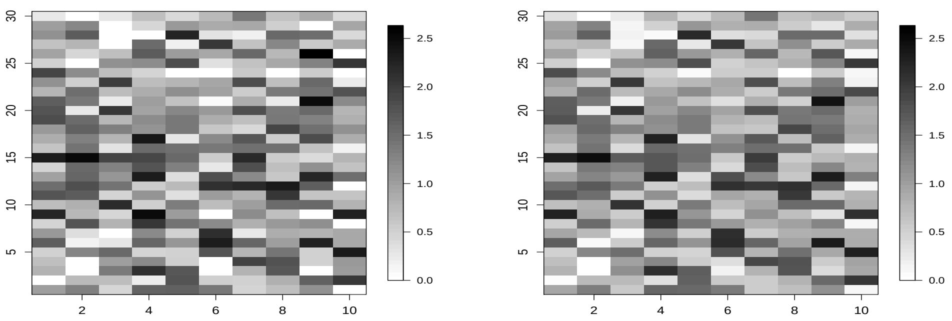

Figure 3: Image plots for true $\beta_{i,j}$ values (left panel) and posterior means of the $\beta_{i,j}$s (right panel).

Discussion

The additional simulations evaluate the performance of the model for extreme cases. In the third simulation, all properties belong to a common cluster, while sites belong to one of several clusters. The results suggest that the model is parsimonious and does not induce unnecessary clusters that are not supported by the data. Scenario 4 was constructed so that the effect of each property on amino acid substitution rates is different. Simulation study 5 was more extreme as both the effect of each property and each site was different. This is the most challenging scenario for our model, as our prior favors the clustering of properties. The fact that the reconstruction of the regression coefficient matrices is reasonably close to the true values suggests that the model, in spite of allowing for a potentially very large number of parameters, will not overfit the data.
